# Educational aspects of rare and orphan lung diseases

**DOI:** 10.1186/s12931-021-01676-1

**Published:** 2021-03-24

**Authors:** Tiago M. Alfaro, Marlies S. Wijsenbeek, Pippa Powell, Daiana Stolz, John R. Hurst, Michael Kreuter, Catharina C. Moor

**Affiliations:** 1 Unit of Respiratory Medicine, Coimbra Hospital and University Centre, Coimbra, Portugal; 2 ERS Early Career Members Committee Representative for Assembly 12: Interstitial Lung Diseases, Lausanne, Switzerland; 3 Department of Respiratory Medicine, Erasmus Medical Centre, University Hospital Rotterdam, Rotterdam, The Netherlands; 4 European Reference Network on Rare Lung Diseases (ERN-LUNG), Frankfurt am Main, Germany; 5 ERS Secretary of Group 12.01-Idiopathic Interstitial Pneumonias, Lausanne, Switzerland; 6 European Lung Foundation, Sheffield, UK; 7 Clinic of Respiratory Medicine and Pulmonary Cell Research, University Hospital Basel, Basel, Switzerland; 8 ERS Immediate Past Education Council Chair, Lausanne, Switzerland; 9 UCL Respiratory, University College London, London, UK; 10 Centre for Interstitial and Rare Lung Diseases, Pneumology and Respiratory Care Medicine, Thoraxklinik, University of Heidelberg, Heidelberg, Germany; 11 German Centre for Lung Research, Heidelberg, Germany; 12 ERS Chair of Group 12.01-Idiopathic Interstitial Pneumonias, Lausanne, Switzerland; 13 ERS Elect Early Career Members Committee Representative for Assembly 12: Interstitial Lung Diseases, Lausanne, Switzerland

**Keywords:** Rare diseases, Medical education, Patient education, Medical societies, Delayed diagnosis, Patient participation

## Abstract

People with rare lung diseases often suffer the burden of delayed diagnosis, limited treatment options, and difficulties in finding expert physicians. One of the reasons for the delay in diagnosis is the limited training for healthcare practitioners on rare diseases. This review explores the main concerns and needs for education on rare lung diseases from the perspectives of both patients and professionals. Despite the increasing interest in rare lung disorders and some recent breakthrough developments on the management of several diseases, healthcare professionals, including general practitioners and hospital workers, receive little education on this topic. Nonetheless, many healthcare professionals show much interest in receiving further training, especially on diagnosis. Patients and families want easier access to high-quality education materials to help them manage their own disease. Well-educated patients are better equipped to deal with chronic diseases, but patient education can be challenging as patients’ individual health issues, and diverse backgrounds can create significant barriers. Raising more awareness for rare lung diseases and further development of patient-centred international expert networks like the European Reference Network on Rare Lung Diseases (ERN-LUNG), which includes both experts and patient representatives, are essential for improving care and education on rare lung diseases. Initiatives such as the Rare Disease Day, have been successful in increasing awareness for rare conditions. The development of online tools for accessing information has had positive effects and should be further supported and extended in the future.

## Introduction

In Europe, a disease is defined as rare having a prevalence of less than 1:2000. In the United States of America (USA) a rare disease is one that affects less than 200,000 people nationwide, which is equivalent to around 1:1600 people [1]. Some of these conditions have a much lower prevalence, and are thus called ultra-rare, but there is no accepted definition for this subgroup [2].

The term “orphan diseases” is used for ‘neglected’ conditions, i.e. where research on diagnosis and treatment is sparse, resulting in a lack of effective treatments. The concepts of orphan and rare diseases are different however, as some common diseases may still be considered orphan, because they affect mostly low-income countries and there may be low financial incentives to research them [3].

An important concept about rare diseases is that as a group they are common, and every physician ends up caring for some patients with rare diseases. There are 6000 to 8000 known rare diseases and syndromes. Some estimates suggest that 3.5 to 5.9% of the global population suffers from a rare disease, which translates into 30 million in Europe and 263 to 446 million people affected globally at any point in time [4]. In Australia, the total number of people living with a rare disease is estimated to be 8% of the population, similar to the population living with asthma or diabetes [5]. Even with a conservative estimate that 5% of these have lung involvement, it is likely that over a million people in Europe are affected by a rare lung disease [4, 6, 7]. About 80% of rare diseases are genetically predisposed, and may present at birth or during early life, but some may not present until adulthood, such as alpha-1-antitrypsin deficiency (AATD) and lymphangioleiomyomatosis (LAM). Rare lung disorders are mainly chronic and idiopathic. Some diseases are limited to the lungs, but many have systemic origin or multiorgan manifestations. Pulmonary disease may involve any of the respiratory systemic components, such as the interstitium, alveoli, large and/or small airways, blood vessels or pleura.

Rare diseases are challenging for patients, physicians and researchers [8]. General physicians and clinicians in community hospitals may have less experience with rare diseases, which can lead to a delay in diagnosis and referral to expert centres. A European survey found that in a quarter of patients with rare diseases it took 5 to 30 years from initial symptoms to a confirmatory diagnosis. Forty percent had received an erroneous or no diagnosis. Many patients had to consult several doctors before a final diagnosis was obtained. These delays were mainly caused by unfamiliarity of physicians with rare diseases [9, 10]. A delay in diagnosis can result in prolonged symptoms, disease progression, impaired quality of life, unnecessary medical interventions and admissions, inappropriate pharmacological and non-pharmacological treatment, and perhaps even a higher mortality [11].

Patients struggle to find physicians that have experience with their condition, leading to treatment delay. Expert centres are usually located at large university hospitals, which may require long and expensive travel for appointments. As there is still limited development of effective treatments for rare lung disease, even after reaching a correct diagnosis, ineffective or incorrectly dosed treatments are sometimes initiated. This may increase the treatment burden and risk of side-effects. This is exacerbated by the frequent lack of robust evidence, for example from randomised controlled trials. The low prevalence and awareness for rare diseases creates many challenges for funding and access to clinical research, and makes it imperative to foster cross-country collaboration [8]. Due to the small number of patients, trials are often multinational and expensive. Hence, education on rare diseases is also important for the general public and policy makers, as more awareness leads to more funding possibilities and facilitates access to research projects. Reimbursement policies are often variable between regions, which can cause further difficulties for accessibility of newer drugs to patients. This can lead to a sense of abandonment by affected patients.

Challenges for clinicians include difficulty in developing experience on conditions that they encounter rarely. Clinicians may not be aware of available expert networks and therefore experience problems in referring these patients. It can be more difficult to be adequately trained in these diseases, and there are few opportunities to bring new knowledge into practise afterwards. Most medical schools, and education for other healthcare professionals, do not have a specific rare disease program or module for under-graduates [11, 12]. This stems from the notion that doctors are not expected to have knowledge on all 8000 rare diseases and that future physicians will rarely encounter a patient with any of these diseases. Hence, medical students are taught to think of common diseases as the explanation for uncommon presentations: “When you hear hoofbeats, don’t expect to see a Zebra” [13]. Hence, one of the objectives for training programs should be that clinicians consider a diagnosis of a rare disease when faced with a specific cluster of suggestive symptoms [14, 15]. Some medical faculties have built programs focusing on rare diseases with success [16].

### Which educational initiatives have been established in Europe?

The European Union (EU) approved an action on rare diseases in 2009, stating that “rare diseases are a threat to the health of EU citizens insofar as they are life-threatening or chronically debilitating diseases with a low prevalence and a high level of complexity”. This action on rare diseases highlighted the importance of supporting “adequate education and training for all health professionals to make them aware of the existence of these diseases and of resources available for their care” [17]. The EU has since championed many important initiatives on the diagnosis and management of rare disorders.

The first European Reference Networks were established in 2017, and included the ERN-LUNG, a European Reference Network on Rare Respiratory Diseases [18] The ERN-LUNG is a non-profit, international, patient-centred and scientific network that is committed, Europe-wide and globally, to the prevention, diagnosis and treatment of rare respiratory diseases through patient care and advocacy, education and research. The ERN-LUNG has nine core networks which represent the diversity of diseases and conditions that affect the lungs. These include cystic fibrosis, primary ciliary dyskinesia, non-cystic fibrosis bronchiectasis, pulmonary hypertension, interstitial lung disease (ILD), chronic allograft lung dysfunction, mesothelioma, AATD and other rare lung diseases [19]. A list of the main rare lung diseases is presented in Table 1. Other networks may care for patients who have primary disease elsewhere, but where lung manifestations are common (e.g. granulomatous lymphocytic interstitial lung disease, GLILD). Currently, the network includes 60 healthcare providers form 12 different European countries. As well as the nine disease subgroups, the network is organized in nine Functional Committees, one of which is Professional Training & Continued Medical Education [20].

**Table 1:** List of some of the rare lung disorders

Rare lung disease
α1-antitrypsin deficiency
Cystic fibrosis (CF)
Non-CF bronchiectasis
Primary ciliary dyskinesia (PCD)
Interstitial lung diseases (ILD)
Idiopathic pulmonary fibrosis (IPF)
Sarcoidosis
Connective tissue diseases
Lymphangioleiomyomatosis (LAM)
Birt–Hogg–Dubé syndrome
Eosinophilic pneumonia
Pulmonary alveolar proteinosis
Adult pulmonary Langerhans cell histiocytosis
Granulomatous lymphocytic interstitial lung disease (GLILD)
Other interstitial lung diseases
Pulmonary hypertension
Pulmonary arterial hypertension
Chronic thromboembolic pulmonary hypertension
Chronic lung allograft dysfunction
Bronchiolitis obliterans syndrome (BOS)
Restrictive allograft syndrome (RAS)
Mesothelioma
Other rare lung diseases

The European Respiratory Society (ERS) launched a project called Harmonised Education in Respiratory Medicine for European Specialists (HERMES) which promoted the harmonization of the postgraduate training of specialists in respiratory medicine throughout Europe. One outcome of the project was a core syllabus which contains a mandatory module on orphan lung diseases, assuring that specialists that undertake the HERMES examination do have training on these diseases [21, 22]. More recently, the ERS has developed a continuing professional development programme, supporting lifelong learning for pulmonary specialists. Several orphan and rare lung diseases have been covered in this plan [23]. The ERS has also established Clinical Research Collaborations (CRCs), which are European multi-centre networks of researchers aimed at advancing science in a specific lung disease, involving many different stakeholders, including researchers from other specialities, funding agencies and patients. A number of CRCs have been established in rare diseases, such as the European Granulomatous-Lymphocytic Interstitial Lung Disease Network, and projects in AATD, PCD, IPF, childhood ILD (ChILD), pulmonary hypertension, and bronchiectasis [24].

The European Lung Foundation (ELF) was founded by ERS in 2000 and aims to bring together patients and the public with respiratory professionals, in order to positively influence lung health. The ELF has developed a wide variety of education initiatives. For example, they provide over 40 online factsheets in 27 languages about common as well as rare lung diseases, to ensure equal access to high-quality education for patients worldwide [25, 26]. The ELF has also developed Patient Advisory Groups in some of the key rare lung diseases to ensure that patients can drive the development of the materials that they need about their lung condition. An example of this is the ELF Patient Priorities websites (including ones on bronchiectasis, ChILD, LAM and sarcoidosis).

The French National Plan for Rare Diseases 2018–2022 recognizes the need for improving the education of healthcare professionals and aims to develop training modules on rare diseases via health simulation and theoretical teaching modules in the field of genomics, as well as creating rare disease research professional career paths [27]. The online resource Orphanet was established in France by the French National Institute for Health and Medical Research in 1997, but became a European endeavour from 2000, supported by grants from the European Commission [28]. Orphanet has gradually grown to a Consortium of 40 countries, within Europe and across the globe. Online resources can be useful for clinicians and are recommended as tool to make a difficult diagnosis [29].

### What are the educational needs of healthcare professionals?

**Fig. 1:**
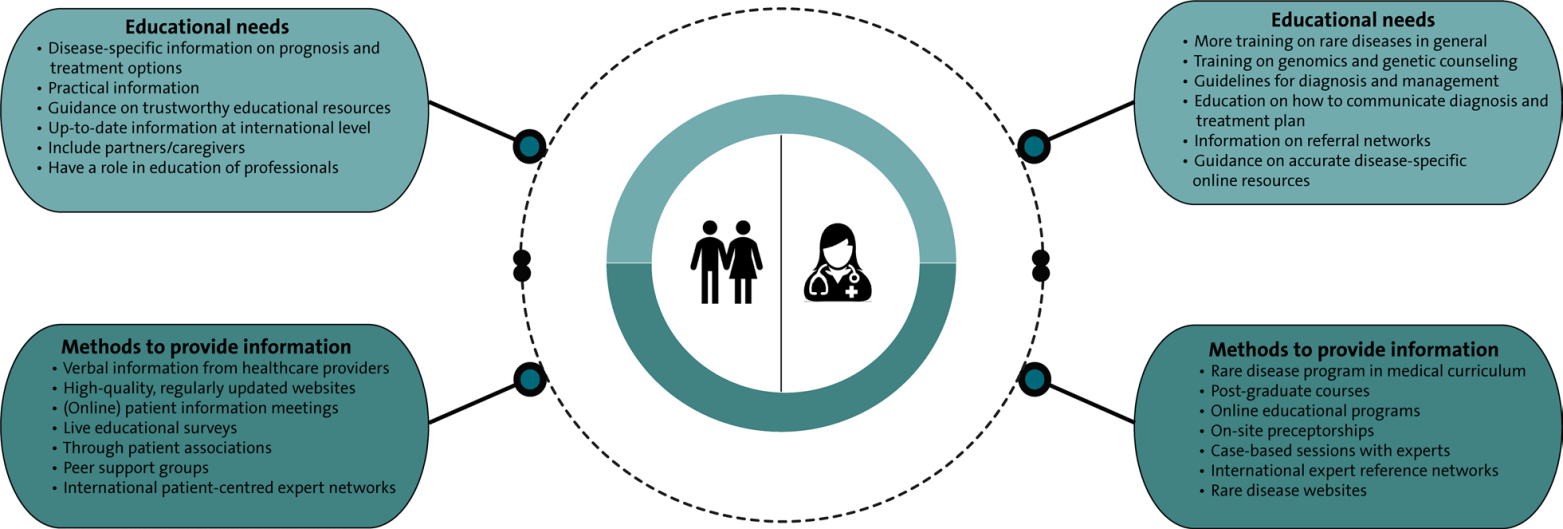
Main education needs and methods to provide information on rare lung diseases for patients and health care workers

The diagnosis and care pathway of patients with a rare lung disease often starts at the primary healthcare or general practitioner (GP) level. These professionals stand at the frontline of the health care network but mostly deal with more common diseases. The ERS created an educational task force responsible for investigating the awareness of European GPs about rare diseases and developing educational materials that can help primary care and GPs dealing with these patients. They developed a Delphi survey on adult and one on paediatric rare lung diseases and distributed it among GPs. Most respondents had over 1000 patients under their care, and 41% followed patients with a rare lung disease, most commonly sarcoidosis, cystic fibrosis, idiopathic pulmonary fibrosis (IPF), AATD and LAM. The surveys found a lack of general knowledge on the diseases that were questioned. The authors argued that the creation of a user-friendly website for GPs and other primary care workers could be useful to raise more awareness for rare lung diseases [30]. In pulmonary hypertension, state-of-the art summary publications, management algorithms and guidelines, online educational programmes, and on-site preceptorships in expert centres have been mentioned as important aspects of education for healthcare professionals [31]. Further proposed forms of education resources in other rare lung diseases are postgraduate courses, webinars, case-based sessions, and expert forums [32] (Fig. 1).

There is little research on the educational needs for rare diseases among healthcare professionals. A recent study in Spain surveyed 169 physicians, including 132 from primary care and 37 from hospitals, and found that half of hospital-based physicians had received postgraduate training on rare diseases while in primary care only 18.9% did. A similar proportion of both groups (40.9% in primary care and 45.9% in hospitals) had attended continuous medical education (CME) events on rare diseases in the previous 5 years. The main reported challenges were the lack of diagnostic guidelines, delay or inability to make a definite diagnosis and doubts about referral. Thus, there is a specific need for dedicated training activities focusing on disease presentation and discussing when to/where to refer patients for further diagnostic workup. All of the respondents displayed interest in receiving further training, especially on genetic counselling, and wished to receive more information on specific rare disease websites [12]. Another study found that the vast majority of medical students felt that their knowledge about rare diseases was not sufficient. Due to this lack of knowledge, students were afraid that they would not be able to serve the needs of patients with rare diseases in the future [11]. Healthcare professionals, both GPs as well as physicians in hospitals, that were trained on rare diseases felt more confident caring for patients with these disorders [12, 33].

A 2017 US survey on the educational needs for IPF among hospital healthcare professionals received 182 responses (156 from physicians). The highest ranked educational need was on how to reach the diagnosis of IPF. Participants expressed the need for printed or online educational materials, and support staff for educating patients [34]. In a European-wide survey about gaps in care for pulmonary fibrosis, distributed through the ERN-LUNG network, less than half (39%) of participating healthcare professionals responded that they received education on how to communicate information on diagnosis and treatment plan with their patients [35]. One of the challenges for physicians managing rare diseases is that patients or family members can sometimes become experts on their own disease, knowing more about it than professionals [36].

### What are patients’ needs for education on rare lung diseases?

Patient education is a valuable component of disease management, and may enhance shared decision making and communication, increase concordance with treatment, reduce anxiety, and help patients to feel more in control [37]. Educated patients are more likely to use healthcare services in a responsible way and obtain higher benefits from treatment [38, 39]. Moreover, educated patients are more independent and empowered for dealing with a chronic debilitating disease, such as rare lung disease. In fact, studies on the supportive care needs of caregivers and patients with pulmonary fibrosis show that the most frequently reported needs are information and education on disease progression and disease management [35, 40]. Nevertheless, many patients feel that they receive incomplete information, both before and after diagnosis [41].

Improved patient education should thus be a part of any successful medical program for rare lung disease. However, educating patients is more challenging than educating healthcare professionals, as the patients’ own health issues, diverse backgrounds, and language can create significant barriers [42]. Furthermore, the complex aetiology and heterogeneity of some diseases make it even more difficult to provide accurate, tailored disease education to patients [37, 43]. A personalized approach, taking into account the individual’s goals, personal preferences, cultural beliefs and lifestyle is needed [26]. It is also important to realize that patient’ informational needs may change during the disease course, and thus, regular re-assessment of patient’s needs and perspectives is crucial [44] (Fig. 1).

There are few studies on patient’s education needs in lung diseases. The majority of the available resources are directed at common respiratory diseases, such as chronic obstructive pulmonary disease (COPD) [45]. Most literature about educational needs in rare lung diseases focused on ILD, especially IPF [45–47]. Patients with ILD reported different needs than patients with COPD. A survey in the USA found that patients ranked disease progression and what to expect, as well as IPF drug therapy, followed by IPF diagnosis as the main educational needs [34]. Literature on how to provide information and education to patients with rare lung diseases is scarce. Moreover, patient opinions on the best way to receive education may differ; some patients prefer verbal information, others written information, but many patients would like to receive a combination of both [41]. Although there is accurate information available online, most information is outdated, of modest quality, or only available in English [48–50]. Currently, there are no criteria for patients to identify which online resources are trustworthy [49]. Therefore, it has been proposed to develop quality standards to label accurate, high-quality online educational resources [50]. Two studies in IPF and sarcoidosis found that live patient information meetings seem a good way of educating patients and their caregivers [51, 52]. An interactive voting system with direct projection of responses was used to interview and educate patients. The vast majority of patients highly appreciated this, indicating that this may be a promising method to enhance patient education.

The ERN-LUNG involves patient input in all activities and has at least one Patient Advocacy Group representative in all disease areas and Functional Committees. The Professional Training & Continued Medical Education Committee developed a survey in 2018 on the educational needs of both healthcare professionals and patients. Most patients responded that there was a need for education at a European level. The most important barriers on accessing education were language, cost of travel and health limitations for travelling. The majority favoured online resources and meetings, but 60% welcomed face-to-face meetings. Almost all respondents thought that patients should be involved in the education of professionals [32]. In a survey among healthcare professionals caring for patients with pulmonary fibrosis, 65% answered that their hospital offers educational activities, such as face-to-face patient information meetings, support groups, eHealth programmes, and education sessions led by specialist nurses; 39% of surveyed patients was involved in any of these activities [35].

Patient advocacy groups continue to be an important source of education and support for patients, for example through organising patients support groups and providing disease-specific information and education [31, 53]. Moreover, representatives of patient organizations often have the opportunity to visit (international) conferences, such as the ERS annual conference, in order to provide their patients with up-to-date and evidence-based information.

### How can we increase awareness of rare lung diseases in the general public?

Public awareness about rare lung diseases is crucial to enable early diagnosis and treatment and may help to support patients and their families in daily life [54]. Patients with rare lung diseases often experience misunderstanding because of the general unawareness of their disease [51, 52]. Hence, increasing awareness of rare lung diseases among the general public has been mentioned as important unmet needs of both patients and healthcare providers [31, 35, 53].

The Rare Diseases Day was created in 2008 by Rare Diseases Europe, a non-governmental patient-driven alliance of patient organisations representing 944 rare disease patient organisations in 73 countries [55]. It takes place at the last day of February each year. The campaign has grown into a global event, involving over 100 countries in 2019. There are other successful campaigns, such as the rare disease week in USA. In Spain, the government declared 2013 as the Spanish Year of Rare Diseases. During the last decade, patient organizations of individual rare lung diseases or disease groups have also initiated yearly awareness periods. For example, an annual World Pulmonary Hypertension Day is organized every year, and September has been designated as Pulmonary Fibrosis Awareness Month [31, 56]. These initiatives will hopefully further increase awareness for specific rare lung diseases.

Recently, members of the European Parliament (MEPs) established a MEP lung health group, which explores both legislative and non-legislative options to promote lung health and prevent respiratory disease at a European level. This group is supported by ERS and ELF, and focuses on increasing funding, advocating for patients suffering from lung diseases, and ensuring equal access to care involving all different stakeholders. Several European patient organisations for rare lung diseases, such as CF, pulmonary hypertension, AATD, and IPF are members of the MEP lung health group. Initiatives like this are important to allow rare lung disease to get much needed visibility with policy makers [57].

### What changed with the COVID-19 pandemic?

COVID-19 has been referred to as a once in a century health crisis, and will probably have a profound and lasting effect on healthcare and society in general, including medical education [58]. The EURORDIS network used the rare barometer survey programme to study the effects of the pandemic on European patients with rare diseases [59]. Between 18th of April and 11th of May there were 6945 responses from 36 countries, including 865 responses from cystic fibrosis patients out of 1187 with Rare Pulmonary diseases. The majority (83%) reported severe disruptions on their care. Out of these, 60% lost access to diagnostics, 60% were unable to receive therapies and 80% had interventions such as physiotherapy postponed or cancelled [60, 61]. However, half of respondents participated in online consultations, 90% found online education tools designed to help them manage the disease and 80% felt that the pandemic strengthened their family unit. Despite the obvious challenges, the COVID-19 pandemic has also accelerated digital ways of care and enhanced self-monitoring. This can stimulate patient empowerment, facilitate insights in disease course, and improve access to information and low-threshold communication with the hospital via online consultations [62].

The pandemic has also led to major disruptions in undergraduate and postgraduate education; nevertheless, there was a fast-paced transition to online teaching and further use of simulated virtual patients [63–66]. The ERS International Congress, as well as many other international and national training and scientific events became online events in 2020 and for the foreseeable future. Online events can improve patient participation as the need for travel is reduced. In 2021, the first European IPF conference, organized by the patient organization EU-IPFF, and the first bronchiectasis patient conference will take place online. On the other hand, many patients may still prefer real-time local meetings in their own language. Thus, it will be important to maintain a balance between face-to-face and online meetings in the coming years.

One of the most successful responses to the pandemic was the development of a multitude of online training events such as webinars and online courses, both for healthcare workers and for patients. The increasing use of virtual simulation may be a good opportunity for education on rare lung diseases. These initiatives will hopefully be preserved after the pandemic. The rapid development of COVID-19 vaccines may also serve as a framework for faster development of new medicines for rare lung diseases [67, 68].

### What are priorities for the future?

Transnational collaborations are needed to improve education in rare lung diseases. This will allow for larger multi-centre patient registries and clinical trials, which will hopefully lead to increasing insights in specific rare lung diseases and development of new therapies [69]. The medical community and policy makers have shown a growing interest in rare lung diseases in the last years. Many countries have developed a national rare disease program, and education is a vital component of these plans. It is important that different actors in the education field, such as ERS and ERN-Lung, increase synergies (e.g. working on a common platform). There were outstanding scientific developments in the last decade in several disease areas, such as pulmonary hypertension, IPF and LAM. These were only made possible by collaborative efforts of researchers, clinicians, governments, pharmaceutical industry, patients, and patient organizations [15].

One of the main challenges for the future is to shorten the diagnostic delay. The best chance to improve this is by increasing physician awareness for rare lung diseases, and perhaps more importantly educating them on how to get help and access expertise for difficult cases. This expertise can be accessed, for instance, through reference networks such as the ERN-LUNG initiative.

Patients and families have repeatedly expressed the need for more accurate disease-specific information, but also concerns on the quality of available online educational resources. It is also important to continue developing and improving websites such as orpha.net and the ELF patient priorities websites, which provide patient-led guidance on subjects that matters most to patients affected by rare lung conditions. Patients and patient representatives are now included in expert networks, and their actions and influence will hopefully benefit all those affected with rare lung diseases. Besides, patient advocacy groups will likely play an increasingly important role in educating and supporting patients and their families.

## Conclusions

Patients with rare and orphan lung diseases often suffer meaningful delays before getting a definite diagnosis, which is partially related to a lack of knowledge and unawareness of both healthcare professionals and the general public.

Patients need more accurate information on the diagnosis, prognosis and management of their condition. During the last years, international networks and websites have been created to provide up-to-date online information for patients. The COVID-19 pandemic accelerated the implementation of online education, both for patients as well as for healthcare professionals. These advances may facilitate patient access to educational events, even after the pandemic.

The interest in rare lung diseases has grown exponentially in the last few years, in parallel to breakthrough discoveries on the pathogenesis and management of rare lung diseases. International collaboration and networking are not only important for care and research, but are also critical for improving education for rare lung disorders.

## Data Availability

Data sharing is not applicable to this article as no datasets were generated or analysed during the current study.

## Abbreviations

AATD: α1-Antitrypsin deficiency
BOS: Bronchiolitis obliterans syndrome
CF: Cystic fibrosis
CME: Continuous medical education
COPD: Chronic obstructive pulmonary disease
CRC: Clinical Research Collaboration
ELF: European Lung Foundation
ERN: European Reference Network
ERS: European Respiratory Society
EU: European Union
GLILD: Granulomatous lymphocytic interstitial lung disease
GP: General practitioner
HCP: Healthcare practitioner
HERMES: Harmonised Education in Respiratory Medicine for European Specialists
ILD: Interstitial lung disease
INSERM: French National Institute for Health and Medical Research
IPF: Idiopathic pulmonary fibrosis
LAM: Lymphangioleiomyomatosis
MEP: Member of the European Parliament
PAH: Pulmonary arterial hypertension
PCD: Primary ciliary dyskinesia
RAS: Restrictive allograft syndrome
USA: United States of America
